# The Theory of Reasoned Action as Parallel Constraint Satisfaction: Towards a Dynamic Computational Model of Health Behavior

**DOI:** 10.1371/journal.pone.0062490

**Published:** 2013-05-03

**Authors:** Mark G. Orr, Roxanne Thrush, David C. Plaut

**Affiliations:** 1 Department of Epidemiology, Columbia University, New York, New York, United States of America; 2 Department of Psychology, Carnegie Mellon University, Pittsburgh, Pennsylvania, United States of America; French National Centre for Scientific Research, France

## Abstract

The reasoned action approach, although ubiquitous in health behavior theory (e.g., Theory of Reasoned Action/Planned Behavior), does not adequately address two key dynamical aspects of health behavior: learning and the effect of immediate social context (i.e., social influence). To remedy this, we put forth a computational implementation of the Theory of Reasoned Action (TRA) using artificial-neural networks. Our model re-conceptualized behavioral intention as arising from a dynamic constraint satisfaction mechanism among a set of beliefs. In two simulations, we show that constraint satisfaction can simultaneously incorporate the effects of past experience (via learning) with the effects of immediate social context to yield behavioral intention, i.e., intention is dynamically constructed from both an individual’s pre-existing belief structure and the beliefs of others in the individual’s social context. In a third simulation, we illustrate the predictive ability of the model with respect to empirically derived behavioral intention. As the first known computational model of health behavior, it represents a significant advance in theory towards understanding the dynamics of health behavior. Furthermore, our approach may inform the development of population-level agent-based models of health behavior that aim to incorporate psychological theory into models of population dynamics.

## Introduction

Some of the most successful models of individuals’ health behavior are variants of the reasoned action approach. This success is due to: a moderate degree of variance in behavior can be accounted for by its constructs [1], [2], [3]; it applies across several key health behaviors to include drinking [4], exercise [5], substance use [6], health screening [7], and sexual risk [8]; and, interventions that change its constructs can, in fact, generate change in behaviors (see [9] for a review).

The Theory of Reasoned Action (TRA) [10] is the embodiment of the reasoned action approach (also see the Theory of Planned Behavior [11], and the Integrated Model, [12]). Here, behaviors are driven directly by intentions towards a behavior. Intentions are driven directly by attitudes and perceived norms related to the behavior. Attitudes and perceived norms are formed from beliefs. For example, an attitude towards performing a behavior is represented in the equation below, where (b) is belief strength (the subjective probability that the outcome of a behavior will come true), (e) is an evaluation of the outcome associated with the behavior (the valence) and, the summation sign captures the aggregation across beliefs:
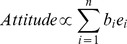



Beliefs have a special status in that they are foundational in forming attitudes and perceived norms and are the only inroad to changing attitudes, perceived norms and, ultimately, intention [13].

Although the TRA was not designed to capture changes in beliefs, research on the application of the TRA has provided evidence that beliefs can change in systematic ways (e.g., the desirability of an outcome related to a behavior), which can, in turn, change attitudes and intention [14]. In short, the TRA provides a psychologically grounded model of the belief – intention link, but without much theoretical attention to how beliefs are formed or changed, except to say that intentions and attitudes can change through changes in beliefs. Thus, the TRA remains a relatively static theory of health behavior and behavioral change.

A completely different approach to understanding health behavior comes from the field of social networks. This work posits that the health behavior of individuals is dynamic and sensitive to social context. In short, people affect others’ behavior. This idea is well supported empirically [15], [16] and is implicated in several areas of health behavior, e.g.: smoking cessation [17], mental health [18], emotions [19], suicidal ideation [20], drug use [21], and obesity [22]. The social network literature, however, does not address the possible psychological mechanisms involved in the spreading of behavior across social network ties.

In this article, we put forth an extension of the TRA that can accommodate the dynamic nature of health behaviors suggested by the social networks literature and that is also captured, to some extent, by the evidence of intention change in the TRA. To this end, we turn to methods and theory from both personality theory and the more recent literature on attitudes for which there is substantial work on the dynamics of attitude formation and change.

The recent advances in the basic theory of attitude formation and change have integrated theory from cognitive science into a dynamic theory of attitude representation and process [23], [24]. This work, using a computational theory called constraint satisfaction, conceptualizes an attitude as a distributed memory representation across a set of interconnected beliefs (constraint satisfaction is described in detail below). By this account, an attitude is a state in a dynamic system that is reconstructed from a set of inputs. The inputs, which represent the immediate social context, can activate beliefs in memory. The interconnections between beliefs represent more long-term attitude structure–these are learned slowly though exposure to many social contexts over time. Personality theory has addressed a similar set of issues, using constraint satisfaction, relating to how one’s behavior is influenced by social contexts and situations [25], [26], [27], [28]. Here, behavior is considered in tandem with a person’s situational context. To explain behavior one must know not only the structure of the personality but also the contexts in which behavior operates.

From these two related literatures, the major import for health behavior is that attitudes and behavior are at once context-sensitive (based on the cues and inputs in the environment) and stable (due to the learned interconnections among the beliefs and cognitions in the system). This, to our mind, fills in the theoretical lacuna with respect to the dynamics of health behavior.

The model of the TRA we put forth here is directly concerned with understanding the dynamics of intention formation and change. Our model affords intention formation and change that is at once sensitive to the immediate context and also stable across a set of similar contexts–i.e., intention is dynamically constructed from an individual’s learned pre-existing belief structure and the beliefs of others in the individual’s social context Specifically, our model considers intention as a distributed memory representation across a set of beliefs. To borrow from Conrey and Smith [23]: Intention is a state, not a thing.

### The Theory of Reasoned Action as Parallel Constraint Satisfaction

We’ve re-conceptualized the Theory of Reasoned Action as a parallel constraint satisfaction system. In the abstract, this type of system represents generic psychological constructs (e.g., features, beliefs, thoughts, units of memory) as set of processing units each of which can vary in its activation level. Each unit represents a hypothesis about whether or not, or how strongly, a psychological construct is activated. Constraint satisfaction refers, in part, to the fact that each unit’s activation level is constrained by the activation levels of other units. For example, if unit X is expected to be active when unit Y is active, the connection between them should be excitatory. In the same way, if the constraint is such that unit X is not expected to be active when unit Y is active, then there should be an inhibitory connection between them. With no expectation, the connection should be neutral, which effectively means there is no constraint between units X and Y. External inputs to the system (e.g., a social situation or context) have similar effects. If a relevant feature is present in the external input, it will constrain a unit’s activation to be on. In general, for each new input to the system, a constraint satisfaction network settles into a state–via a relaxation procedure–in which the constraints are well satisfied, at a local level. This mechanism has proven successful in both cognitive science and personality and social psychology [29], [30].

**Figure 1:**
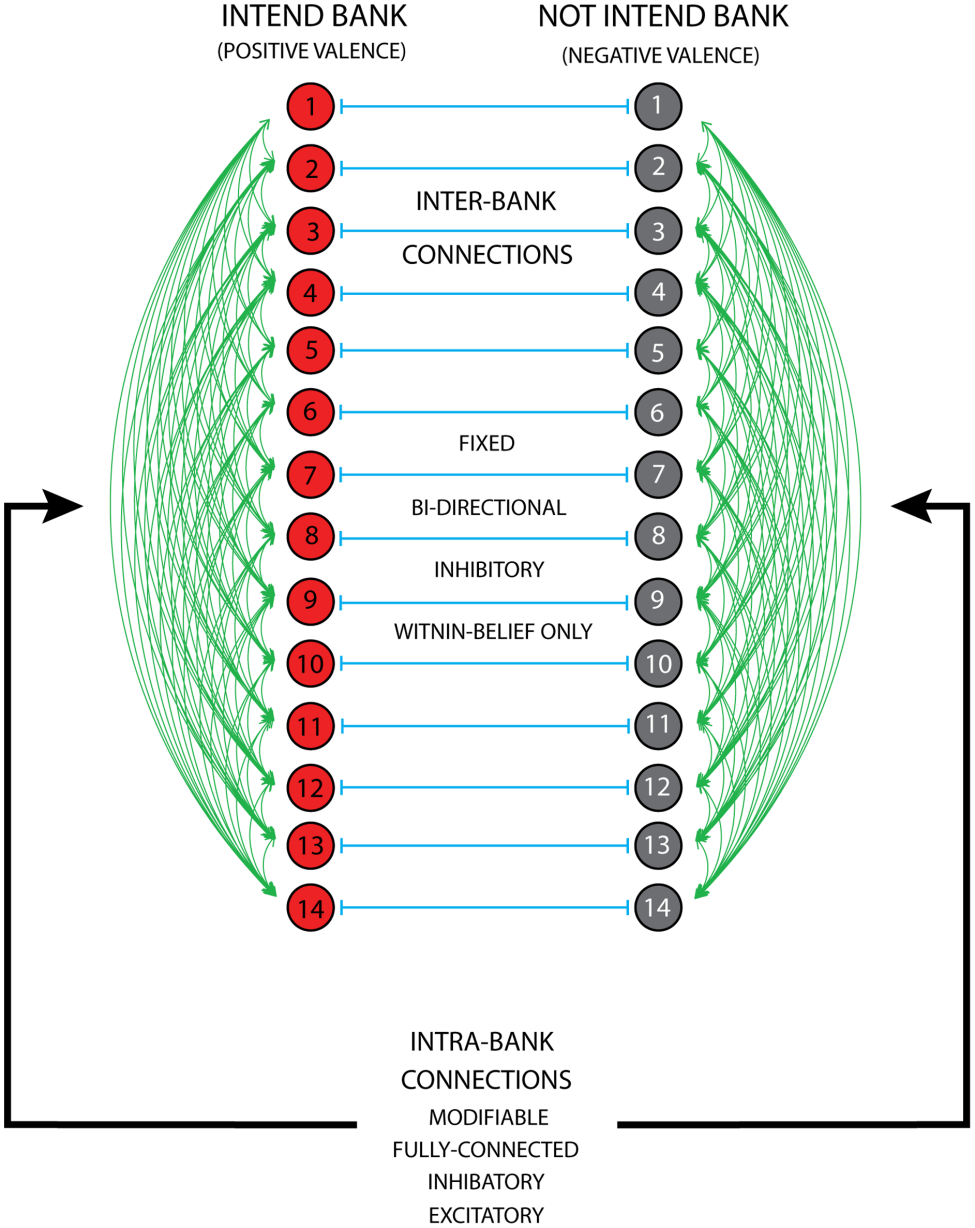
The Theory of Reasoned Action as a constraint satisfaction system. Each belief is split among two processing units; one to capture positive valence (red circles) and the other to capture negative valence (grey circles). The numbers within each unit index the belief (1 = belief one…14 = belief fourteen). *Inter-bank connections:* The constraint between valence units within a belief is always inhibitory (see the blue connecting lines between units). There are no constraints between units that are both a different belief and different valence. *Intra-bank connections:* Within each valence bank, the beliefs are fully connected (i.e., each belief can constrain all other beliefs of the same valence (see the curved green arrows next to each valence bank). These constraints, called *internal processing constraints,* are modifiable, through learning, from a set of input patterns which represent past social context/situations regarding others’ beliefs. *Dynamics:* External input to the system (not shown here) directly activates the belief units and represents others’ beliefs (called *external processing constraints*). The state of the system at any time point is a function of both the internal and external processing constraints; a constraint satisfaction algorithm dictates the specific form of this function.

Figure 1 illustrates our re-conceptualization of the TRA. In our model, beliefs are the constituents of what we call the intention system. To capture valence, each belief is split between two units; one represents positive valence (to intend) and the other represents negative valence (to not intend); there is an inhibitory connection between them, i.e., each valence of a belief constrains the other valence of the same belief to be less active.

Intention, in TRA parlance, is represented by the pattern of activation across the belief units. It is useful to organize the pattern of activation as two separate banks of intention units as depicted in Figure 1; the intend bank represents intending to do the behavior in question (positive valences of the beliefs, in red), the not intend bank represents not intending to do the behavior (the negative valences, in gray). Thus, at any point in time, the intention of the system is roughly in one of three states: intend (on average the intend bank is more active), not intend (the compliment of intend), ambivalent (where both banks are nearly equally active).

By design, the constraints among beliefs are not pre-specified because there is no a priori reason to do so. These constraints capture what is learned by the system about health behavior from past social contexts or situations through modification of the strength and sign (inhibitory or excitatory) of the constraints. We call these *internal processing constraints* to capture the idea that what is learned represents the stable internal *psychological structure* of a person–a pre-existing belief structure for any social context that is encountered. We assume learning about health behavior occurs over the long-term from exposure to social contexts such as cultural sources, media outlets, family, and friends.

An external input to the system represents constraints on the units from the immediate social context–i.e. others’ beliefs activate the belief units in the system. Inputs are dictated to follow the same valence structure that is present in the model. Thus, if the input showed evidence that the positive valence of Belief 1 was active in the social context, the input would constrain the positive valence to be active and provide no constraint on the negative valence of Belief 1. We call these *external processing constraints* to capture the idea that the immediate, short-term social context can constrain the activation levels of the system at any time-point. Furthermore, the degree to which the external inputs constrain the units is controlled by a clamping factor. Conceptually, clamping represents the weight a person gives to others’ beliefs–stronger clamping reflects more weight to others’ beliefs.

The external inputs to the system play a role in both the learning of internal processing constraints and the immediate external constraints on the system. As we will explain below, learning is not always “on” in the system. When it is “off” the external constraints only play a role in the relaxation procedure to determine the current state of the system; when “on” the external constraints have the additional function of potentially leading to a modification of the internal processing constraints.

### Conceptual Overview of System Simulations

The general design of our simulations was to first generate a set of internal constraints (via learning) for a specific, definable context and then to test how the model behaved when embedded in several equally specific and definable external contexts (defined by a set of input patterns). The degree to which (strength of) the external context impinged on the system (the clamping factor) was systematically varied within the simulations.

In the first two simulations, the design attempted to pit learned aspects of behavioral intention against both the nature of and strength of the immediate social context with respect to the same behavioral intention. The nature of short-term social contexts is an analog for exposure to what other beliefs people hold; clamp strength is an analog for how much influence or weight a person gives to the short term context.

In the third simulation, the design attempted to show that our conceptualization of intention, as a pattern of activation in the system, actually mapped onto empirical measures of intention. Thus, we tested the predictive validity of our model against participants’ reported intention.

## Methods

### Ethics Statement

The human subjects data was collected under approval from the Institutional Review Board of the University of Washington. Written informed consent was obtained from all parents of the participants (all were students in school) prior to Year I and Year IV of the study. Furthermore, written assent was obtained from all participants every year of the study. The data used in this manuscript was striped of all identifying information and we did not have any access to the coding scheme between the de-identified data and the larger dataset. Thus, there was no potential way for us to link the individual data points in the data we used with the actual participants.

### Participants & Measures

We used self-report survey data specifically designed to measure the TRA with respect to sexual behavior in adolescents to construct a portion of the inputs for our simulations. These data were part of a seven year longitudinal in-school survey from 1992 to 1998 [31]. The survey sampling methods, demographics, and questionnaire are described in the online Supporting Information (see File S1).

The TRA measures were included in year six of the survey (N = 749, 10^th^–12^th^ grades); we used the outcome and normative belief measures for all simulations. The outcome beliefs captured the following concepts (framed by the question “Do you think having sexual intercourse will make you…”): feel good, be more popular, feel loved, feel experienced, get an STD, get pregnant, regret it later, get HIV, and have emotional stress. These beliefs were defined as the product of the likelihood of occurrence of the outcome (from 1 to 4 where large means more likely) and the evaluation of the outcome if it occurred. The evaluative component captured the valence of the belief (ranging from −2 = very bad, 2 = very good, with a zero midpoint representing neutral). Normative beliefs were defined as the product of the respondent’s perception of a referent’s attitude towards he/she having sexual intercourse and the respondent’s motivation to comply with said referent (the latter ranged from 1 to 4 where large means more likely to comply). The referents were parents, best friend, other friends, favorite teacher, closest sibling. The valence of normative beliefs was captured by the referent’s attitude, ranging from −2 = referent does not think its ok for respondent to have sex to 2 = referent thinks its ok (with a zero midpoint). Thus, the resultant 14 beliefs (nine outcome and five normative) were measured on an integer scale from −8 to 8 (including a zero mid-point); negative indicated negative valence, positive indicated positive valence; magnitude was captured by the absolute value of the scale. See the online Supporting Information (File S1) for details on and examples of these measures.

In one of the simulations (Simulation III) we also used a direct measure of intention (Empirical Intention): “When you are in [next grade], do you think you will have sexual intercourse?” [1 = NO!, 2 = no, 3 = yes, 4 = YES!].

We isolated the following sub-populations of the sample based on gender, school grade (10^th^ or 12^th^), and virginity status (virgin or not virgin): Female, 10^th^ grade, virgin, N = 105; female, 12^th^ grade, virgin, N = 66; and female, 12^th^ grade, not virgin, N = 73.

### Model Specification

Figure 1 represents the general structure of our model–fourteen beliefs separated into 28 units. This is an auto-associator neural network [32]. Not shown in Figure 1 is the existence of two separate banks of ten hidden units each of which were fully projected to and from the respective intention bank (i.e., bi-directional connections to the respective bank). Units were never self-connected; all were connected to a bias unit. The belief units functioned as both input and output units. We implemented a modified logistic activation function across all units. For every input to the system, the units were updated synchronously for nine cycles. A small portion of activation values at the end of each processing cycle for every input pattern was carried over to the initial input for the next input pattern. Activation of the units in the model were restricted to (0, 1).

#### Nature of input patterns

The input patterns used both to generate the internal constraints and to run simulations were of identical structure. The input patterns consisted of a binary vector of length 14 and its bitwise compliment (total length is 28). The first 14 bits on the vector represented the inputs to the intend bank; the others represented the not intend bank.

We constructed six types of *input sets*. Set P25, was defined by a set of input patterns that, on average, had a probability of.25 for representing the positive valence of each belief (independently for each belief). This set was designed to represent the context in which there was a relatively strong inclination for *not* intending to do the health behavior. To represent the contexts of a neutral and a strong inclination for intending to do the health behavior, we constructed Set P50 and Set P75–defined exactly as P25, but with.50 and.75 probabilities in place of.25.

The other three sets were derived from the empirical dataset described under Participants. Sets F10V (Female, 10^th^ Grade, Virgins), F12V (Female, 12^th^ Grade Virgins), and F12NV (Female, 12^th^ Grade Non-Virgins) were transformed from the empirical data on its original scale to fit the structure of the model in a way that preserved the belief valence structure. The original scale for both outcome and normative beliefs was from −8 to 8, including a midpoint of zero. We transformed this scale in two steps at the individual participant level. First, we transformed the original scale to a binary scale by assigning negative values to 0, positive to 1 and zero probabilistically to 0 or 1 (with a probability of.5 to assign as 1). Then we defined the negative valence of the input structure as the logical compliment to the positive valence. In short, we mapped the original scale onto the positive/negative valence structure of the input pattern in a way that preserved the original valence but not magnitude of the scale. Each participant was represented as a single example in her respective input set.

#### Generation of internal processing constraints

The internal processing constraints were of two kinds: 1) the connection weights between intra-belief valences, and 2) the connection weights between the belief units within each valence bank. The former were fixed at −.20 to provide an inhibitory constraint between valences within each belief. For the latter, we generated two sets of internal constraints. The first, using the P25 input set, represented the case in which the model learned from contexts with a strong bias for not intending to do the health behavior (called the P25 weight set). The second, using the F10V input set, was a context in which the model learned the belief structure of Female, 10^th^ grade virgins (called the F10V weight set).

To train the connection weights for a given set of input patterns, we trained the system in batch mode for 200 epochs using the generalized delta-rule. Within each epoch, the error was computed for the 7–9^th^ processing cycle of each input pattern. Clamp strength was fixed at 0.50 (the connection weight from each external input to the system units). We used a single input set of 50 input patterns to generate the connection weights for the P25 weight set. For the F10V weight set, we trained the model using the full data set for female, 10^th^ grade virgins (N = 105).

#### Structure of the empirical input sets

**Figure 2:**
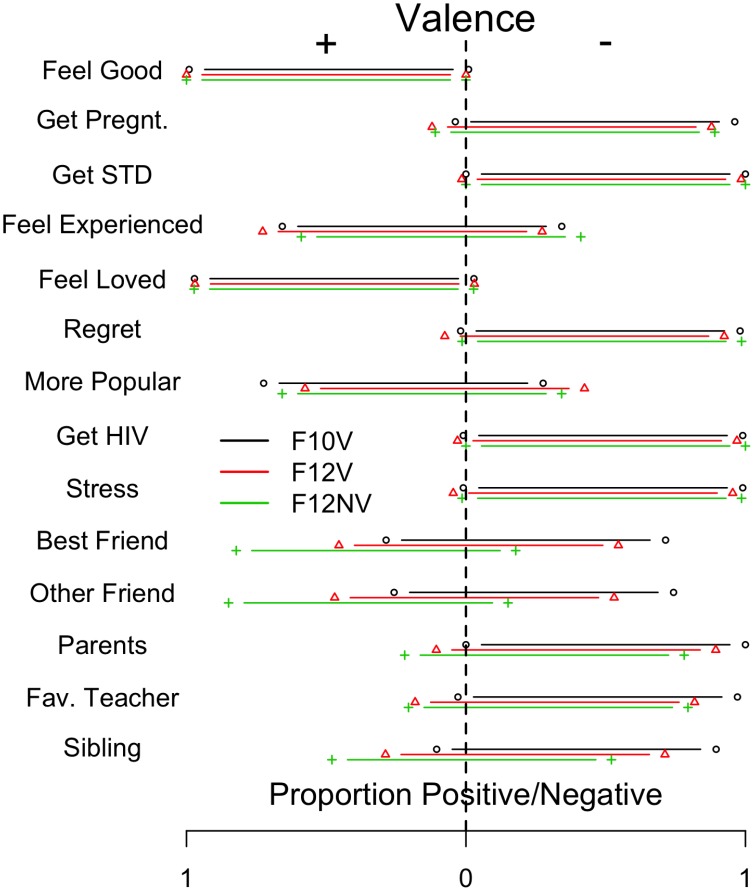
The valence structure in the empirical input sets used to generate the internal processing constraints for Simulation II. Each horizontal line represents the proportion of respondents (in the respective input set) that had a positive or negative valence (the proportion negative is, by definition, 1 minus the proportion positive). Black represents the F10V input set; red and green, F12V and F12NV, respectively. The proportion positive is captured from the zero-midpoint on the x-axis towards the left (see the dotted black vertical line); negative is to the right.

Figure 2 represents the F10V, F12V and F12NV input sets separately, showing the proportion of respondents that had a positive and negative valence for each of the 14 TRA constructs. By considering these proportions as frequencies in the input to the system, we can understand to a rough approximation what the system learned when exposed to the F10V input set. In particular it learned: 1) inhibitory constraints between constructs where one is frequent and the other is not, 2) excitatory constraints between constructs where both are frequent, and 3) no constraint between constructs that are both infrequent. For, example, it should learn an inhibitory constraint between the “feel good” construct and the “get an STD” construct in the intend bank of units in the system. At a more aggregate level, we can understand what the system learned by illustrating the average exposure to valence from the inputs. The mean valence, across beliefs, was 0.29 positive for the F10V input set–it leaned heavily towards the negative valence (to not intend). For comparison, this statistic was 0.36 and 0.42 for the F12V and F12NV input sets, respectively, indicating an increase in positive beliefs about sexual behavior from 10^th^ to 12^th^ grade and between 12^th^ grade virgins and non-virgins.

We describe the structure of the input sets in Figure 2 as a way to understand what might be learned by the neural network model. An alternative way to understand this would be to analyze the connection weight matrix directly. Without hidden units, the weight structure would be relatively straight-forward to analyze. However, due to the number of hidden units in our model, such an analysis would not be fruitful.

#### Nature of external constraints during simulations

External constraints were the input patterns presented to the system during the simulation phase (always after the internal constraints were generated; learning was not allowed during simulation). For Simulations I and II, the input patterns were scaled by the clamping factor (2-levels,.10 and.50, called weak and strong clamping conditions, respectively). For Simulation III, only the strong clamping condition was used.

### Simulations

#### Simulation I

This simulation used the P25 weight set simulated under input sets P25, P50 and P75 (separately, for a set of three input conditions). The clamping factor was varied across the two levels describe above. In short, keeping the internal constraints constant at P25, Simulation I used a 3 (input set) X 2 (clamping factor) factorial design.

#### Simulation II

This simulation used the F10V weight set simulated under input sets F10V, F12V, F12NV, P50 and P75. The clamping factor was varied as in Simulation I, thus this was a 5 (input set) X 2 (clamping factor) factorial design.

#### Simulation method for I and II

For each condition in the simulations, 30 runs were conducted (a run is analogous to an individual person in an experimental setting). Each run consisted of ten epochs of the same 50 input patterns (totaling 500 exposures to inputs). The input patterns across runs were different. Data was collected on the 9^th^ processing cycle for each input pattern (500 data points per run/per condition). Learning was blocked during all simulations.

The input patterns across runs were different for each simulation within a condition. For Simulation I, this was accomplished by simply generating a random set of 50 inputs for each run. For Simulation II, this was accomplished by sampling 50 times (without replacement) from the appropriate input set to create the input patters for each run. For example, for run #1 under the F12V input condition, we sampled the 66 respondents in the empirical sample of Female, 12^th^ grade virgins 50 times without replacement to generate the input patterns for this run.

#### Simulation III

This simulation was different in nature compared to Simulations I and II. The F10V weight set was simulated under the F10V input set, as in simulation II, with a clamping factor of 0.50. However, the simulation consisted of only one run and one epoch per run. That is, we presented the system with 105 separate input patterns only once. Each of the 105 input patterns represented one of the 105 respondents in the F10V subset of the empirical data. Data was collected on the 9^th^ processing cycle for each input pattern, resulting in 105 data points for the simulation, each of which represented the intention formed given the input. As in Simulations I and II, learning was blocked during the simulation.

## Results and Discussion

For each simulation condition in Simulation I and II we present the mean activation of the positive and negative valence banks across the 500 input patterns and 30 runs per condition (1500 data points per mean).

### Simulation I

**Figure 3:**
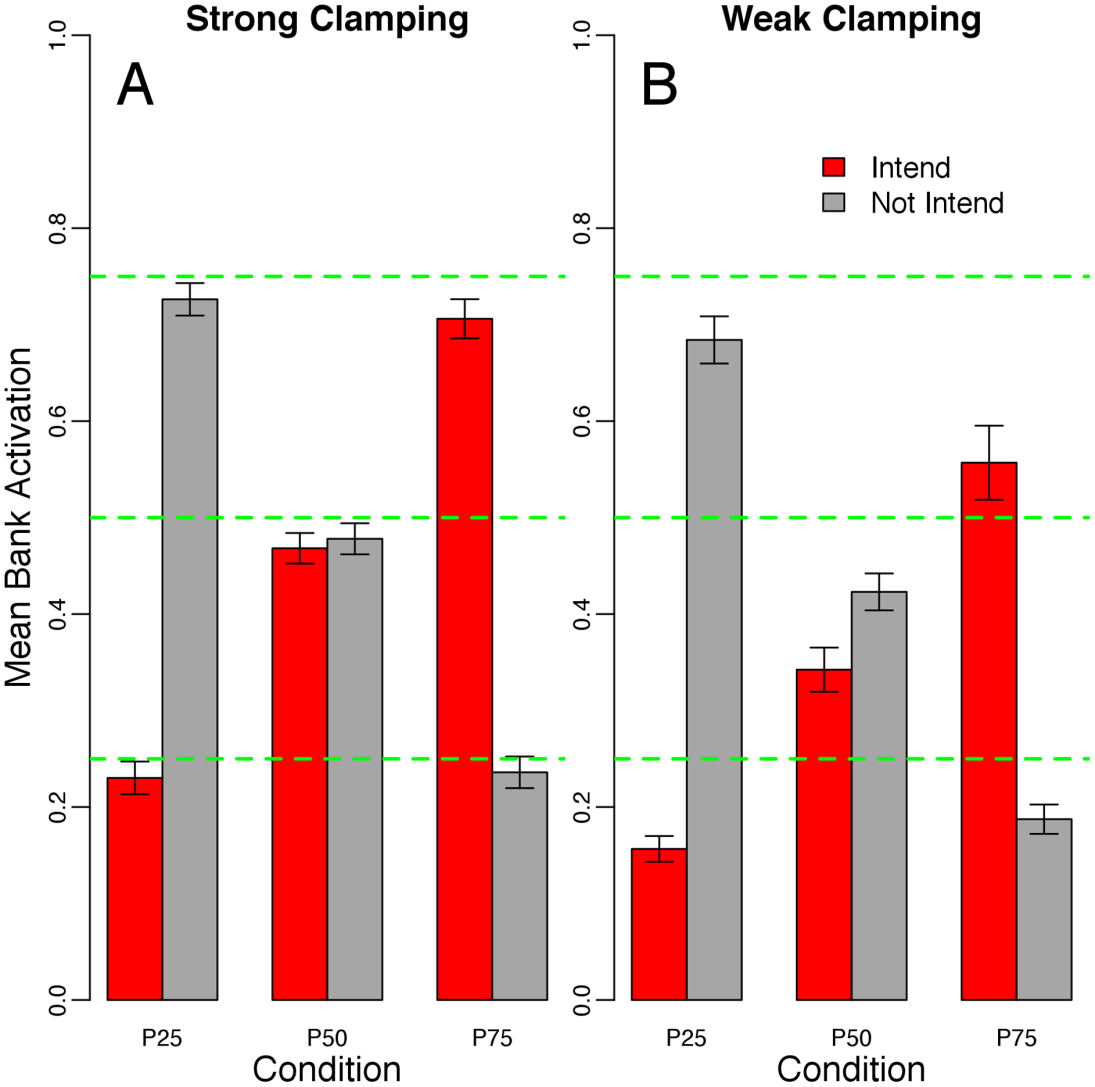
The results from Simulation I. The x-axis shows the three input set conditions: P25, P50, and P75. The y-axis represents the mean activation of each valence bank (red = positive, to intend; grey = negative, to not intend). The green dashed-horizontal lines indicate mean activation levels of 0.25, 0.50, and 0.75. The error bars show the standard deviation across 30 runs of each input set X clamping factor condition (using n = 30 in the denominator). Panel A shows the strong clamping condition; Panel B shows weak clamping.

Figure 3 shows the mean activation for each valence bank. The green dashed-horizontal lines on the y-axis demarcate mean activation levels of 0.25, 0.50, and 0.75 to capture the predicted mean activation values of the valence banks, given that the external constraints dominated the behavior of the system–e.g., for the P25 condition the mean activations should be around 0.25 and 0.75 for the intend and not intend banks, respectively, if the external constraints dominate; for the P50, 0.50 for both banks; for the P75 condition, the reverse of the P25 condition. Thus, the difference between the dashed horizontal lines and the model output illustrates the extent to which the internal constraints affected the state of the intention system.

Figure 3a shows the results for Simulation I under strong external clamping. For the P25 input set, the mean activations of the intend and not intend banks approximated what was predicted if the external constraints were driving the system. Under this condition, the input patterns were highly similar to those used to generate the internal constraints (i.e., the system learned from contexts that strongly favored not intending). Thus, it is difficult to infer the extent to which the system behavior was driven by either the internal or external constraints; the P50 and P75 input sets provided a better test. For these two conditions, the mean bank activations closely matched what would be expected if the internal constraints had a minimal effect on system behavior. In short, under strong external clamping the external constraints (i.e., the short-term social context representing others’ beliefs) dominated the behavior of the intention system.

Figure 3b shows the weak clamping condition. The P25 input set shows that, even under weak clamping, the inputs still had a strong effect on the system. However, the P50 condition indicates that the internal constraints also had an effect on system behavior. The lower activation for the intend bank compared to the not intend bank mapped onto the bias of negative valence (not intend) inherent in the internal constraints. The P75 condition provides further support that the internal constraints played a role in system behavior. Although the intend bank was much more activated than the not intend bank, its activation was much less than the not intend bank in the P25 condition.

In sum, Simulation I suggests that both the internal and external constraints can constrain system behavior– the internal constraints do so only with weak clamping. That is, both the pre-existing learned belief structure and others’ beliefs in the immediate social context were implicated in intention formation.

### Simulation II

Simulation I demonstrated that the system behavior was sensitive to both the internal and external constraints (i.e., learned aspects and short-term social context, respectively). In Simulation II, we tested our theoretical model in a more realistic context.

**Figure 4:**
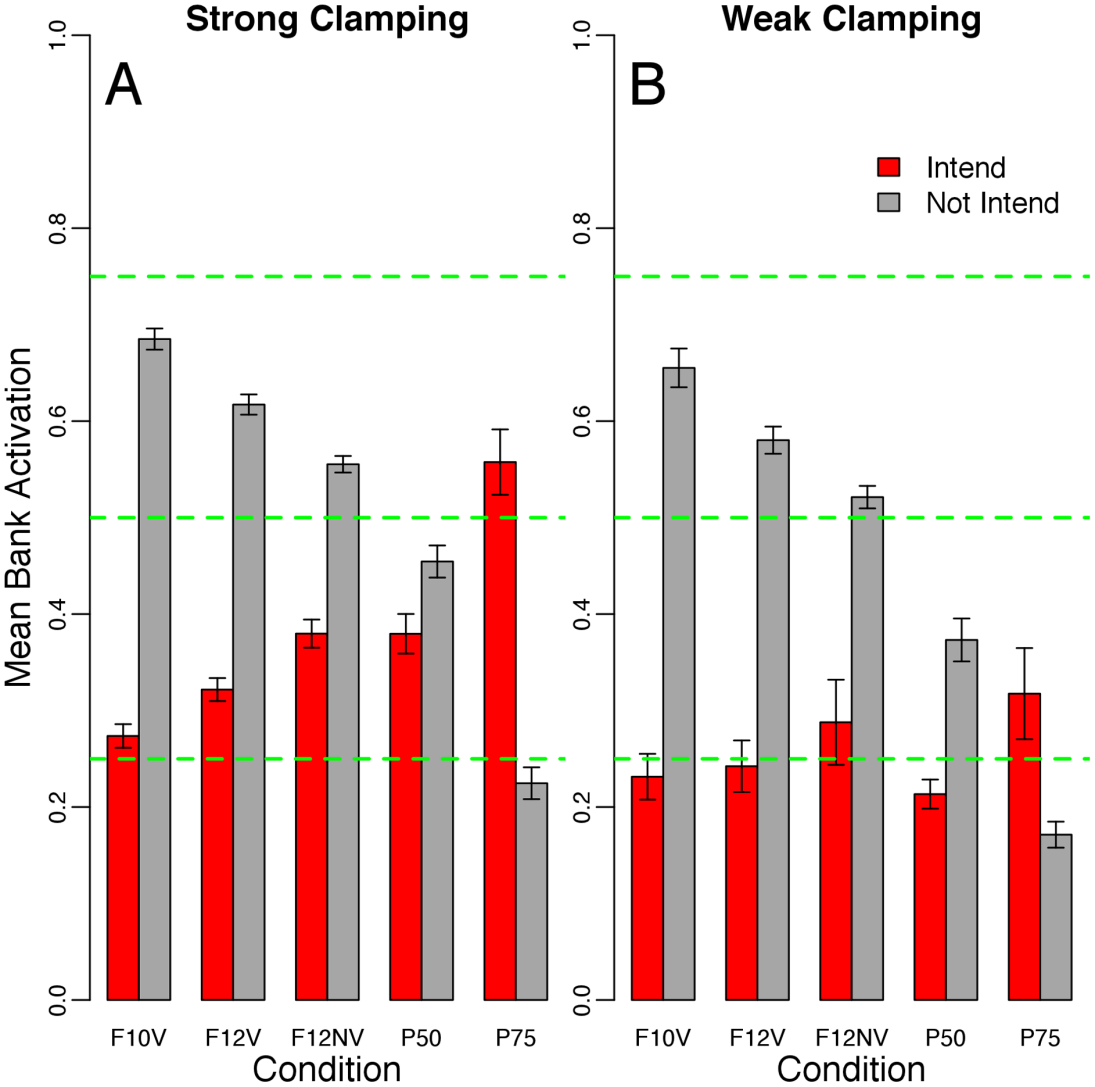
The results from Simulation II. The x-axis shows the three input set conditions: F10V, F12V, F12NV, P50, and P75. The y-axis represents the mean activation of each valence bank (red = positive, to intend; grey = negative, to not intend). The green dashed-horizontal lines indicate mean activation levels of 0.25, 0.50, and 0.75. The error bars show the standard deviation across 30 runs of each input set X clamping factor condition (using n = 30 in the denominator). Panel A shows the strong clamping condition; Panel B shows weak clamping.

Figure 4 has the same structure as Figure 3. For the strong clamping condition, it is clear that the F10V input set strongly activated the system. This was expected because the F10V input set was used to generate the internal constraints. The F12V and F12NV input sets exhibited an increase in the activation of the intend bank and a parallel decrease in the not intend bank, attributable to the increase in positive valance represented in these input sets. Thus, the system was sensitive to the external constraints. However, these input sets do not provide much insight into the degree to which the internal constraints affected behavior. To this end, we presented the P50 and P75 input sets to the system. The P50 input set shows that the system was sensitive to both the internal and external constraints. Both banks were activated less than expected (0.50) and the intend bank was activated less than the not intend bank (capturing the bias inherent in the learned internal constraints). The P75 input set further supports the findings from the P50 condition. The intend bank was much less activated than the expected value of 0.75. In short, under strong clamping, the system showed sensitivity to both the internal and external constraints. This contrasts with the results of Simulation I in which under strong clamping the system did not show much sensitivity to the internal constraints.

The results under weak clamping generally echoed those under strong clamping; the internal and external constraints both played a role in system behavior. However, this condition revealed a further property of the system. The P50 condition showed weaker activation of the intend bank compared to the F10V condition. This suggests that there may have been competition among the units in the intend bank (inhibitory and excitatory constraints cancel out one another), which accords with the structure of the inputs that generated the internal constraints (See Figure 2). Figure 2 illustrates that there should be more inhibitory connections among the units in the intend bank compared to the not intend bank. This was not the case for Simulation I because there was not as much inherent structure in the inputs. The comparison between the weak and strong clamping conditions reveals a related point. When comparing the weak to strong clamping, most of the change in activation is exhibited from an increase in the intend bank activation. This may also stem from competition among the units in the intend bank.

### Simulation III

Simulation III was designed to test how well the model could predict intention in the empirical data set, using the F10V weights and input set. In short, the simulation produced an intention score (called Model Intention) for each of the 105 female 10^th^ grade virgins in the F10V input set. These were compared directly to the empirical measure of intention, called Empirical Intention (described under Participants & Measures). In short, this provides a way to compare intention as defined in the model with intention as defined in the Theory of Reasoned Action.

The data for this simulation was not aggregated as in Simulation I and II. For each of the 105 input patterns tested in Simulation III, we computed an intention score equal to the mean of the intend bank minus the mean of the not intend bank. The intention score had a theoretical range from negative one to one and was continuous in nature; negative values represented not intend; positive values, intend. This measure was characterized statistically as follows: M = −0.413, SD = 0.181, range = (−0.924, 0.086). From this measure, we constructed a four-level categorical variable of intention, called Model Intention, using the following cut-points: (−1, −0.5] = 1, (−0.5, 0] = 2, (0, 0.5] = 3, and (0.5 to 1) = 4. The values of Model Intention were designed to be directly equivalent to the same values of Empirical Intention such that 1 = (NO!), 2 = (no), 3 = (yes), 4 = (YES!).

Table 1 shows the cross-tabulation of Model Intention with Empirical Intention. There was a reasonable correspondence between Model and Empirical Intention. Using Fisher’s Exact Test, the probability of sampling this cross-tabulation, given the marginals, was equal to 0.0004355; in other words, there was a significant relation between the two intention measures. Furthermore, Model Intention miss-matched most for the Empirical Intention levels one and three. We conducted further analysis to test whether slightly shifting the cut-points used to construct Model Intention would improve the correspondence between the two intention measures. This was not possible. Our analysis indicated that there were 27 input patterns at Empirical Intention levels one and two between a very narrow range of the model intention score (in continuous form); specifically, between the values of −0.422 and −0.420. Among these input patterns, however, there was no cut-point for constructing Model Intention that increased the correspondence between Model and Empirical Intention.

**Table 1:** Crosstabulation of the categorical neural network intention measure by the empiricial data set for the female 10th grade virgins (N = 105).

	Intention Categories in Neural Network			
Empirical Intention	1	2	3	4
1 (NO)	**23**	**27**	**0**	**0**
2 (no)	**10**	**26**	**0**	**0**
3 (yes)	**0**	**17**	**1**	**0**
4 (YES)	**0**	**1**	**0**	**0**

Table 2 shows the mean value of the model intention score (the continuous measure) for each of the four levels of Empirical Intention. Table 3 shows the model intention score (continuous) regressed onto Empirical Intention**.** Taken together, these two tables suggest a positive linear relation between the model intention score and Empirical Intention.

**Table 2:** Mean value of model intention score (continuous) for each level of Empirical Intention.

Empirical Intention	Mean	SE	N
1 (NO)	−0.50	0.02	50
2 (no)	−0.39	0.03	36
3 (yes)	−0.22	0.03	18
4 (YES)	−0.29	–	1

**Table 3:** Regression of model intention score (continuous) onto Empirical Intention (dummy-coded).

Empirical Intention*	Coeff.	SE	p< x
Intercept	−0.50	0.02	0.001
2 (no)	0.10	0.03	0.01
3 (yes)	0.27	0.04	0.001
4 (YES)	0.21	0.16	ns

Notes: *reference category was intention = 1 (NO); Adj. R-sq = 0.28; omnibus *F* (3, 101) = 14.39, p<.001.

## Conclusions

The main findings of our modeling effort were twofold: First, Simulations I and II showed that past experience biased the behavior of the system towards an activation state that reflected the internal processing constraints. This was qualified by both the level of clamping (the stronger the clamp strength, the weaker the effect of the internal processing bias) and the structure of the inputs that defined the internal constraints (more structure in the internal constraints results in stronger bias, e.g. Simulation II compared to Simulation I). In terms of health behavior, this implies that the immediate social context (others’ beliefs), although potentially influential, may be systematically constrained by both the weight a person gives to others’ beliefs (clamping) and a person’s pre-existing belief structure (due to learning). This property embodies the central notion of constraint satisfaction–simultaneous mitigation of constraints from multiple sources.

Second, the system’s intention state was predictive of the actual, empirically measured intention scores, as shown in Simulation III. This occurred despite the fact that the inputs for the simulation only captured the valence structure and not the strength of the beliefs (see above regarding how we transformed the empirical data into inputs). And, we used a very simple algorithm to construct Model Intention–subtract negative from positive valence and cut into equally spaced quartiles. The explanation for this predictive power is straightforward. The model learned to represent well the inputs that were presented to it during the simulation. (Remember, the inputs for training of the F10V weight set were identical to the inputs used in Simulation III.) So, upon presentation of an input, it captured well the belief structure which, for the empirical data used in the simulation, correlated with the empirical measurement of intention.

### An Extension of the Theory of Reasoned Action

Is our model really an extension of the TRA? We argue yes for three reasons. First, both our model and the TRA fall under the rubric of the long-standing expectancy-value (EV) model (see [33] for historical review)–in our model and in the TRA, intention is a function the belief expectation (probability of an outcome of a behavior) and the valuation (valence) across a set of beliefs. Second, our model uses the same constructs and measures, but just defines behavioral intention in a different way. Third, as an extension, our model is simply a computational implementation of the TRA. This is an accepted approach to theory development in social psychology [34].

What is gained by virtue of a computational instantiation of the TRA? The primary advance comes from the re-conceptualization of intention. By our theory, intention is a state that arises from constraint satisfaction–a highly non-linear process involving internal and external processing constraints. Intention formation is generated on the fly, dynamically, from the interaction between current social situations and past learning. In other words, dynamics are naturally situated into health behavior theory via constraint satisfaction.

The second advance centers on learning. The TRA is silent with respect to the explicit learning mechanisms that generate a person’s belief structure. However, it is self-evident that both the belief strength (the subjective probability that the belief will come true) and the expectation of the outcome of the belief (the belief valence) must be learned. By our theory, learning operates through changes in the constraints among the beliefs and captures the statistics of exposure to others’ beliefs. This implies that learning captures the distribution of and the higher-order associations among beliefs inherent in the past social experience. It is not clear to what extent this contradicts the TRA because of the dearth of work on learning with respect to it.

The third advance is that in our model the effects of past experience on health behavior, via learning, are considered separate from the effects of direct social influence–separate but fully interdependent as dictated by the constraint satisfaction mechanism. In contrast, although the exact mechanisms of learning in the TRA are not clearly defined, there is no question that beliefs, and thus intention, are meant to represent learning from past experience alone; direct social influence has no role on immediate behavior. In other related theory, however, there is precedent to treat social influence separately from learning or simply as the sole driver of behavior change. For example, the social diffusion of health behavior is typically represented by a influence coefficient [17], [22] or a threshold rule [35]; learning is not considered. In a similar vein, most agent-based models of group norms do not incorporate learning, but capture the group dynamics via social influence [36]. Recent work in personality theory incorporates direct social influence as a primary driver of behavior [28], [37]. The current work casts health behavioral intention as a function of both past-experience and direct social influence.

An important criterion for evaluating a new theoretical advance is the extent to which it makes new, testable predictions. Simulation III showed that our model could predict intention under conditions in which the internal and external constraints were closely aligned–i.e., the model’s past experience was very similar to its current social context. However, what is the prediction under other circumstances, especially those that are crucial for health behavior applications? For example, in the adolescent sexual behavior literature, key transition periods, such as going from primary to secondary school, are very important. How would the model predict intention as it evolves over key developmental periods? Here, it is clear that the predictions for the TRA and our model are different. If past experience and the current social context are different, our model predicts that intention will be a function of the two, via constraint satisfaction. The TRA, however, does not make predictions that are based on both past and current contexts and thus it does not make predictions about such key developmental transitions beyond suggesting that beliefs and thus intention will change over time.

In short, our model is capable of making specific predictions that are based on the similarity of the current context and the past social contexts–key constructs towards understanding developmental transitions, and arguably other types of life-course changes that might be related to health behavior (e.g., changes in contexts that are generated by policy implementation). The central advance in terms of testable predictions, then, is that our model points to the need for understanding both the past and present social contexts to predict changes in intention. This prediction is amenable to experimental procedures or some type of prospective observational studies that have a relatively high-temporal density of measurement (e.g., using repeated measures of social contexts in a social network).

Another type of prediction of our model concerns what is learned from past social contexts. Our model is very specific with respect to what it learns, as described above. The predictions in this regard are equally specific–learning a belief structure will capture the higher-order correlations among a set of beliefs. This is a testable prediction, given that we know what the past social context was. It might be a fruitful area of research in the health behavior field to explore the belief structure–not just what beliefs are salient but the strength of the relations among beliefs–because this may be a strong driver of the dynamics of intention.

Finally, a general set of predictions come from the attitude formation literature. Our model puts the TRA on an equal footing with recent advances in attitude formation, and thus, offers a set of predictions related to the extant theoretic controversies. For example, the debates over implicit/explicit change [38], and constructivist/static memory representations [23] come with empirical predictions. These, we think, might find a way into the TRA as extended by us.

## Summary

In sum, our model advances the Theory of Reasoned Action in meaningful ways that make testable predictions. First, it explicitly models the development of a belief structure via learning from past experience with others’ beliefs. Second, direct social influence–the effect of others’ beliefs in the immediate social context–is captured in the external constraints and is incorporated seamlessly with past experience via constraint satisfaction. Third, intention is conceptualized as arising from a parsimonious and dynamic process among belief valence banks and is predictive of empirical intention measurements. Furthermore, by instantiating our theory in a computational framework, we have opened the doors to multi-agent models of population health that are based on plausible psychological processes.

## Supporting Information

File S1 **Participants and Measures.**
(DOCX)
