# Supplementary material for: The Theory of Reasoned Action as Parallel Constraint Satisfaction: Towards a Dynamic Computational Model of Health Behavior
Source: PLoS One. 2013 May 3;8(5):e62490. doi: 10.1371/journal.pone.0062490 (PMC3643950; doi:10.1371/journal.pone.0062490)
Supplement: File S1 — Participants and Measures. (DOCX) [file pone.0062490.s001.docx]

**Participants & Measures**

**Sampling characteristics of participants.** This was a 7-year longitudinal study of students in grades 3-6 at the initial wave of data in 1992 (funded by the NIH – NIDA (DA07047) and NIMH (MH63274)). Sixteen of 65 elementary schools and 7 of 10 middle schools in the district were invited to participate of which 10 and 3 agreed to participate, respectively. A total of 2319 students were enrolled in the target grade levels in this sample of schools; parental consent was obtained for 1177 (56% consent rate) students; 1173 of these participated in the study. The distribution of race/ethnicity and gender in the sample was similar to the district-wide distribution of the same grades; however, their was a slight bias toward higher income families in the sample. Surveys were administered yearly (spring) by trained interviewers who read the questions aloud while the students followed along using response booklets (each child had their own booklet). Teachers were not present at the time of interview.

The data we used came from waves in the 6^th^ year of the study. In this year, measures of the fully elaborated Theory of Reasoned Action were collected. The sample size for the three cohorts included in our analysis was 749 (89% of the sample). The grade distribution of this sample was 32% in 10^th^, 31% in 11^th^, and 37% in 12^th^; gender was 53% female; race/ethnicity, 47% non-Latino European American, 20% African American, 22% Asian American, and 10% other (to include Latino, Native American, Alaskan Native); family income, 8% < $17,420, 27% between $17,419 and $24,790, and 65% > 24,789; 59% of the respondents never had sexual intercourse.

**Questionnaire items measuring the beliefs for the Theory of Reasoned Action.** This section presents the items used to measure outcome and normative beliefs in the participants described above.

**Outcome Beliefs (9 items) (Higher scores reflect more favorable towards having sex)**

*Each outcome belief was computed as the product of the likelihood and evaluation of each outcome. Therefore, for each item, the respondent was asked about the likelihood and evaluation of each of the 9 outcomes. For likelihood, the question was framed as “Do you think having sexual intercourse will…” [ 1(NO!) to 4(YES!) ]. For evaluation, it was framed as “Do you think the following are good or bad?” [ -2(very bad) to 2(very good) ].

Outcomes presented to the respondents.

1. “feel good”
2. “more popular”
3. “feel loved”
4. “feel experienced”
5. “get and STD”
6. “get pregnant”
7. “regret it later”
8. “get AIDS/HIV”
9. “emotional stress”

**Normative Beliefs (6 items) (Higher scores reflect more favorable towards having sex)**

*Each normative belief was computed as the product of the perception of norms of a referent group and the youth’s motivation to comply with each referent group. Therefore, for each item, the respondent was asked about the referent group norms and motivation for 5 referent groups. For perceived norms, the question was framed as “Does your [referent gp] think it would be OK for you to have sexual intercourse when you are in [next] grade?” [ -2(NO!) to 2(YES!) ]. For motivation to comply, it was framed as “Do you want to do what your [ referent group ] wants you to do?” [ 1(NO!) to 4(YES!) ].

Referent groups presented to the respondents

1. “father”
2. “mother”
3. “best friend”
4. “most of your other friends”
5. “favorite teacher”
6. “the brother or sister you feel closest to”

(*NOTE, we used the average of father and mother if both parents were reported or the value of the reported parent if only one parent was reported so that the computational model only had 5 normative beliefs).

**Calculation of the beliefs in the empirical data.** This section presents, by example, how the valence for the beliefs was calculated.

Example of outcome belief valence calculation (6 examples):


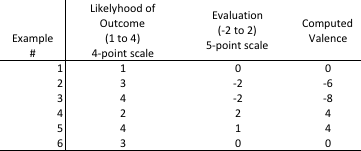


Example of normative belief valence calculation (6 examples):


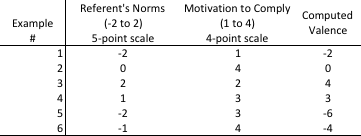


END OF DOCUMENT
